# Signal drift in diffusion MRI of the brain: effects on intravoxel incoherent motion parameter estimates

**DOI:** 10.1007/s10334-024-01183-6

**Published:** 2024-07-13

**Authors:** Oscar Jalnefjord, Louise Rosenqvist, Amina Warsame, Isabella M. Björkman-Burtscher

**Affiliations:** 1 Department of Medical Radiation Sciences, Institute of Clinical Sciences, Sahlgrenska Academy, University of Gothenburg, MRI Center, Bruna Stråket 13, 413 45 Gothenburg, Sweden; 2 Department of Medical Physics and Biomedical Engineering, Sahlgrenska University Hospital, Region Västra Götaland, Gothenburg, Sweden; 3 Department of Radiology, Institute of Clinical Sciences, Sahlgrenska Academy, University of Gothenburg, Gothenburg, Sweden; 4 Department of Radiology, Sahlgrenska University Hospital, Region Västra Götaland, Gothenburg, Sweden

**Keywords:** Repeatability, Quantitative MRI, Preprocessing

## Abstract

**Objectives:**

Signal drift has been put forward as one of the fundamental confounding factors in diffusion MRI (dMRI) of the brain. This study characterizes signal drift in dMRI of the brain, evaluates correction methods, and exemplifies its impact on parameter estimation for three intravoxel incoherent motion (IVIM) protocols.

**Materials and methods:**

dMRI of the brain was acquired in ten healthy subjects using protocols designed to enable retrospective characterization and correction of signal drift. All scans were acquired twice for repeatability analysis. Three temporal polynomial correction methods were evaluated: (1) global, (2) voxelwise, and (3) spatiotemporal. Effects of acquisition order were simulated using estimated drift fields.

**Results:**

Signal drift was around 2% per 5 min in the brain as a whole, but reached above 5% per 5 min in the frontal regions. Only correction methods taking spatially varying signal drift into account could achieve effective corrections. Altered acquisition order introduced both systematic changes and differences in repeatability in the presence of signal drift.

**Discussion:**

Signal drift in dMRI of the brain was found to be spatially varying, calling for correction methods taking this into account. Without proper corrections, choice of protocol can affect dMRI parameter estimates and their repeatability.

## Introduction

Signal drift has been put forward as one of the fundamental confounding factors in diffusion MRI (dMRI) of the brain [1]. Gradual increases or decreases in signal intensity of around 10 percent over a scan time of 15 min have been reported for single dMRI scans of the human brain [2]. In preclinical settings where the scan times can span hours, this becomes an even more pronounced effect to consider [3, 4].

Potential origins of the signal drift in dMRI have been suggested, including B_0_ field drift, flip angle drift, and variable Nyquist ghosting, but a definitive answer is lacking [1, 2]. Dynamic B_0_ field corrections have been shown to reduce the magnitude of the observed signal drift to some extent, but the efficiency varied between vendors and overcompensation was observed in some case [2]. Furthermore, the use of dynamic B_0_ field corrections will increase the overall scan time and can be a source of striping artefacts [5].

Given the unknown origin of signal drift in dMRI and the corresponding difficulty in establishing effective prospective correction methods, a retrospective method building on repeated acquisition of non-diffusion-weighted images (*b* = 0 images) has been proposed [2, 6]. By interspersing *b* = 0 images throughout a dMRI scan, the signal drift can be monitored and quantified. A correction is then applied to all acquired images, also the diffusion-weighted ones. In the original implementation of this correction method, Vos et al. [2] derived and applied the correction on a global scale based on average signal values within a (brain) mask, with a proof of principle in phantom and human brain. This was further developed by Hansen et al. [6] to correct for spatially varying signal drift observed in phantom data. Preliminary data presented by Huynh et al. [7] suggest that these spatial variations also are present in the human brain, but results were based on a single subject and remain to be confirmed.

The implications of signal drift on dMRI are strongly dependent on the targeted biophysical model or signal representation and the accompanying acquisition protocol. An acquisition protocol ordered by *b*-value or diffusion encoding direction will, for example, result in biased parameter estimates, whereas signal drift in data acquired with a less ordered protocol would tend to generate larger residuals and possibly increase estimation variability [2]. This effect can be expected to be particularly pronounced when subtle signal variations at outer regions of the acquisition dimension (e.g. *b*-values) are of interest. Considering and correcting for signal drift is, therefore, likely of high importance, for example, for techniques relying on quantification of signal variations at very low *b*-values, such as intravoxel incoherent motion (IVIM) parameter mapping [8], and at high *b*-values, such as diffusion kurtosis imaging or similar [9].

The aim of this study was to explore the characteristics of signal drift in dMRI of the brain, including spatially varying signal drift, and to evaluate methods for drift correction. The impact of signal drift on dMRI parameter estimates is exemplified for a set of different IVIM protocols.

## Materials and methods

### MR imaging

Ten healthy subjects (age 21–34 years, seven males) were scanned using a 3 T Philips MR7700, software release 5.9.0, with a 32-channel head coil (Best, the Netherlands). The study was approved by the Swedish ethical review authority (Dnr 2020-00029) and all subjects signed informed consent. A subset of the data were included in a previous study evaluating methods for joint IVIM analysis of flow-compensated and non-flow-compensated dMRI data [10].

**Fig. 1:**
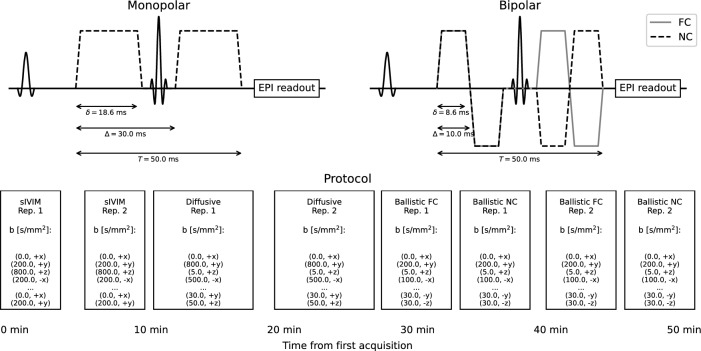
Schematic illustration of the pulse sequences utilized in the study with actual timings displayed (upper left and right) along with the timeline of the full examination (lower panel). The scans with three and ten *b*-values used the monopolar gradient pulses while the scans with seven *b*-values used the bipolar gradient pulses to achieve a combination of flow-compensated (FC) and non-flow-compensated (NC) diffusion encodings. The width of boxes in the lower panel and their separation correspond to the actual scan duration and separation

Three sets of dMRI scans based on a spin echo echo planar imaging sequence were acquired, designed for different types of IVIM analysis (upper panels in Fig. 1): (1) a three *b*-value scan with monopolar diffusion encoding gradients for a simplified IVIM analysis (sIVIM) [8, 11], (2) a ten *b*-value scan with monopolar diffusion encoding gradients for a conventional IVIM analysis assuming that the motion of blood is diffusion-like (pseudo-diffusion; referred to as the diffusive protocol) [8], and (3) two seven *b*-value scans with bipolar diffusion encoding gradients with one designed to be flow-compensated (FC) and the other one non-flow-compensated (NC) for joint analysis of FC and NC data assuming that the motion of blood is ballistic (referred to as the ballistic protocol) [12]. Timings of the diffusion encoding gradients are found in upper left and right panels of Fig. 1. Details about each scan are given below.

All scans were acquired with a prototype pulse sequence enabling use of arbitrary gradient waveforms for diffusion encoding originally developed at Lund University [13] with the following common sequence parameters: TE = 80 ms, TR = 3700 ms, voxel size acquired 2 × 2 × 4 mm^3^ reconstructed to in-plane 1.875 × 1.875 mm^2^, 1 mm slice gap, SENSE = 1.9 (anterior–posterior direction), fat suppression by SPIR and gradient reversal. Dual readout was used for mitigation of Nyquist ghosting [14]. Six diffusion encoding directions ($x,y,z, - x, - y, - z$) were used for all diffusion-weighted scans (*b* > 0), while six repetitions were used for *b* = 0 to acquire the same number of scans regardless of *b*-value. The acquisition scheme was designed by looping through all combinations of *b*-values and diffusion encoding directions $\left( {b_{i} ,d_{j} } \right)$, with $d_{j} \, \in \,\left\{ {x,y,z, - x, - y, - z} \right\}$, with simultaneous increments in both *i* and *j* to distribute acquisitions with the same *b*-value but different encoding direction over the scan time (see examples in lower panel of Fig. 1). All scans were acquired in a single session and each scan was repeated without moving the subject.

#### MR imaging: sIVIM protocol

The sIVIM protocol utilized monopolar diffusion encoding gradients using the three *b*-values 0, 200, and 800 s/mm^2^ with the number of repetitions of each *b*-value obtained from a Cramer-Rao Lower Bound-based optimization resulting in 2, 3 and 1 repetitions of the respective *b*-values for the given scan time set to be 4.5 min [15]. These repetitions were implemented as the *b*-value sequence 0, 200, 800, 200, 0, 200 s/mm^2^, repeated six times as described above to acquire data for all diffusion encoding directions.

#### MR imaging: diffusive protocol

The diffusive protocol utilized monopolar diffusion encoding gradients and a sequence of ten *b*-values similar to what is commonly seen in the literature: *b* = 0, 5, 10, 20, 30, 50, 100, 200, 500, and 800 s/mm^2^. The *b*-values were acquired in a “low–high” order i.e. *b* = 0, 800, 5, 500 etc. Total scan time was 7.5 min.

#### MR imaging: ballistic protocol

The ballistic protocol utilized two scans with bipolar diffusion encoding gradients (FC and NC) with seven *b*-values: 0, 5, 10, 20, 30, 100, and 200 s/mm^2^. The *b*-values were acquired in a “low–high” order i.e. *b* = 0, 200, 5, 100 etc. Total scan time was 10.5 min.

### Preprocessing of data

**Fig. 2:**
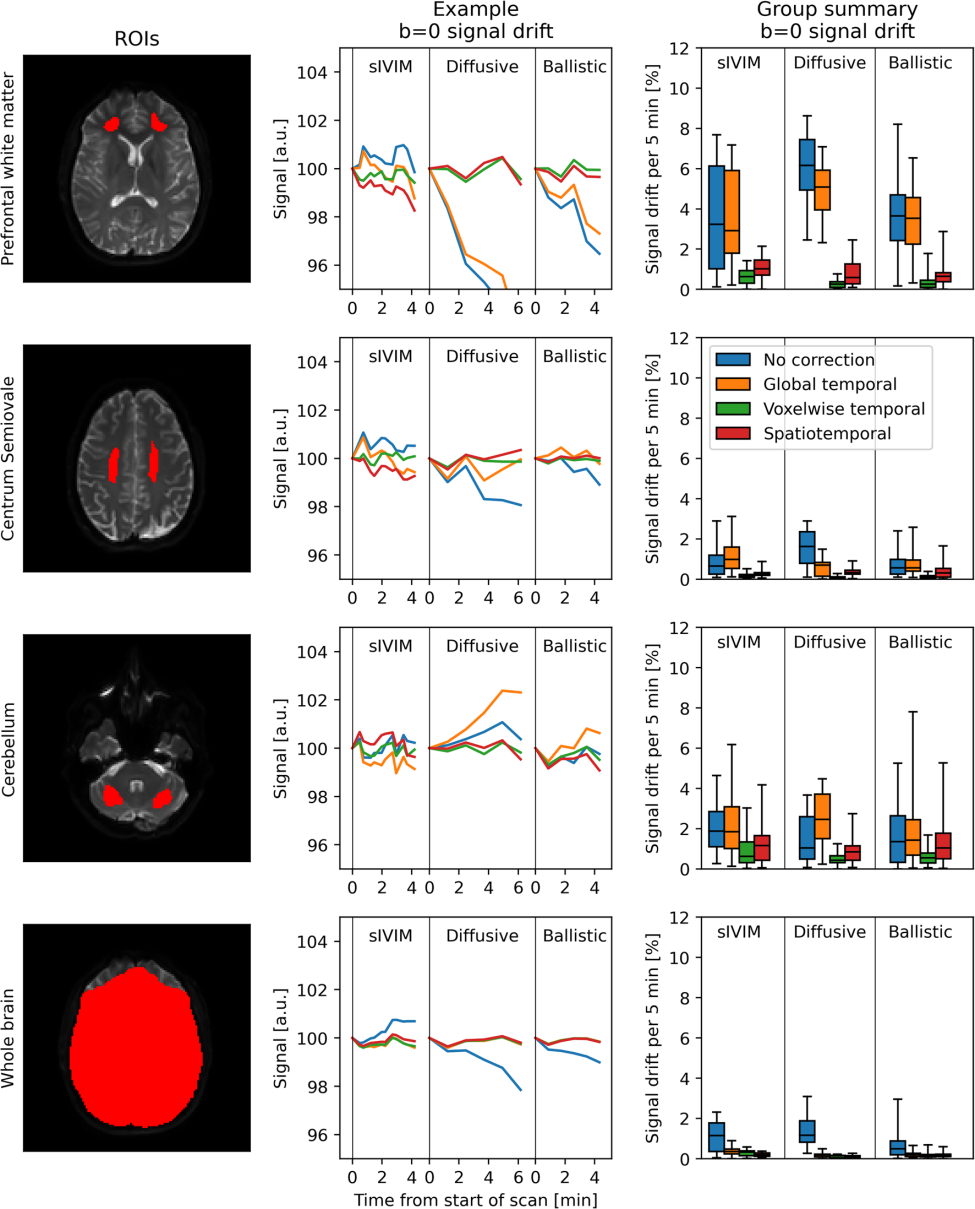
*b* = *0 signal drift.* The left column illustrates the investigated regions of interest (ROIs) for an example subject. The middle column shows how the average *b* = 0 signal, normalized to 100 for the first data point, fluctuated during the acquisition of an example scan from each protocol for the same example subject as in the left column. The colors correspond to different correction methods (see legend in right column) and each section corresponds to a given protocol. The right column shows group summaries where displayed data is the ROI median signal difference between each scan’s first and last *b* = 0 acquisition aggregated over all scans for a given protocol. Boxplots show min, 25th percentile, median, 75th percentile, and max.

To minimize spatial misalignment due to motion and eddy currents, and to get all images to a common space, all images were registered to the first *b* = 0 image of the examination (first *b* = 0 image of the first acquisition of the sIVIM protocol was originally called mono3b, was the naming was changed during the review process. The instance was missed in the revision.) using affine registrations (eddy_correct script in FSL v. 6.0.4) [16]. Non-brain tissue was masked out by brain extraction (BET in FSL v.6.0.4) [17]. Three regions of interest (ROIs) were manually delineated in prefrontal white matter, centrum semiovale, and cerebellum for subsequent ROI-based analyses (examples in Fig. 2).

### Signal drift correction

Three methods for signal drift correction were utilized: (1) a global temporal correction as first suggested by Vos et al. [2], (2) a voxelwise temporal correction corresponding to this global method [6], and (3) a spatiotemporal correction as suggested by Hansen et al. [6].

The temporal corrections fitted a second order polynomial to the *b* = 0 signal as a function of time where the global method fitted a single polynomial to the median signal in the brain, while the voxelwise method fitted one polynomial for each voxel:

1 $$S\left( {n|b_{n} = 0} \right) = k_{0} + k_{1} n + k_{2} n^{2} ,$$

where $S(n|b_{n} = 0)$ is the signal at acquisition $n$ given that it is a *b* = 0 acquisition, and $k_{i}$ are the polynomial coefficients to be estimated. The polynomial coefficients were estimated by finding the least squares solution to data. The correction was then applied to all data, regardless of *b*-value, with corrected signal values calculated as:

2 $$S_{{{\text{corr}}}} \left( n \right) = \frac{S\left( n \right)}{{\hat{k}_{0} + \hat{k}_{1} n + \hat{k}_{2} n^{2} }},$$

where $S_{{{\text{corr}}}} \left( n \right)$ is the corrected signal at acquisition $n$ and $\hat{k}_{i}$ are the polynomial coefficients estimated from the *b* = 0 signal, i.e. the subset ${{\{\ }}n{|}b_{n} = 0\}$.

The spatiotemporal correction followed a similar procedure, but did instead fit a second order polynomial in both time and space:

3 $$S\left( {n;x,y,z | b_{n} = 0} \right) = p_{0} \left( {x,y,z} \right) + p_{1} \left( {x,y,z} \right) \cdot n + p_{2} \left( {x,y,z} \right) \cdot n^{2} ,$$

where $p_{i} \left( {x,y,z} \right)$ is a polynomial containing all zeroth, first and second order combinations of the spatial coordinates *x*, *y*, and *z*, resulting in a total of 81 polynomial coefficients to be estimated. $p_{i} \left( {x,y,z} \right)$ can be found in Appendix A [6]. To avoid overfitting to outliers or data points with high leverage, the polynomial coefficients were estimated by bisquare regression as suggested by Hansen et al. [6, 18]. Before fitting this polynomial to data, a voxelwise normalization to the first *b* = 0 image was applied to remove the underlying anatomical signal distribution in the *b* = 0 images. As for the temporal corrections, the corrected data was calculated as:

4 $$S_{corr} \left( {n;x,y,z} \right) = \frac{{S\left( {n;x,y,z} \right)}}{{\hat{p}_{0} \left( {x,y,z} \right) + \hat{p}_{1} \left( {x,y,z} \right) \cdot n + \hat{p}_{2} \left( {x,y,z} \right) \cdot n^{2} }},$$

where $\hat{p}_{i}$ are the polynomials estimated from the *b* = 0 signal, i.e. the subset ${{\{\ }}n{|}b_{n} = 0\}$.

### Simulation of acquisition order

To study the effect the order in which data are acquired has on derived model parameters, data with acquisition protocol ordered by *b*-value (ordered protocol) were generated, from which parameter maps were derived and compared with those obtained from the actually acquired data (mixed protocol). Data corresponding to the ordered protocol were generated by reordering data corrected with the spatiotemporal polynomial correction based on *b*-value, such that an increasing *b*-value order was obtained with all encoding directions acquired in direct succession. This is in contrast to the mixed protocol used for acquisition of data where *b*-values are mixed and the different encoding directions for a given *b*-value were distributed over the scan time. Following the reordering, the inverse spatiotemporal polynomial correction was applied to simulate the signal drift that previously had been corrected for. This enabled comparison of uncorrected data from the original mixed protocol and uncorrected data from a simulated ordered protocol as well as their corresponding parameter maps.

### IVIM parameter estimation

Before parameter estimation, all scans were directionally averaged with a geometric average to mitigate potential effects of background gradients [19, 20]. To remove potential scaling differences between the FC and NC scans from the ballistic protocol, the two scans were each normalized by dividing with the median *b* = 0 signals within the brain mask before being combined into a single data set. Specifically tailored analysis approaches were then used to obtain a set of IVIM parameters from each protocol, as described below.

#### IVIM parameter estimation: sIVIM

Data from the sIVIM protocol were used to estimate the IVIM parameters *D* and *f* via a segmented algorithm [8, 21]. First, a monoexponential signal representation was fitted to data corresponding to the two non-zero *b*-values:

5 $$S\left( b \right) = Ae^{ - bD} .$$

Since only two *b*-values were used, analytical solutions could be used to first calculate the diffusion coefficient *D* as:

6 $$D = \frac{{\ln S_{1} - \ln S_{2} }}{{b_{2} - b_{1} }},$$

where *S*_*i*_ is the signal at *b*-value *b*_*i*_. The intercept term *A* was then calculated as:

7 $$A = S_{1} e^{{b_{1} D}} .$$

In a second step, the perfusion fraction *f* was calculated as:

8 $$f = 1 - \frac{A}{{S_{0} }},$$

where *S*_*0*_ is the signal at *b* = 0.

#### IVIM parameter estimation: diffusive regime

Data from the diffusive protocol were used to estimate the IVIM parameters *D*, *f,* and D* of the diffusive regime by a least squares fit to data with the following biexponential signal representation:

9 $$S\left( b \right) = S_{0} \left( {\left( {1 - f} \right)e^{ - bD} + fe^{{ - bD^{*} }} } \right),$$

where D* is the pseudo-diffusion coefficient [8].

#### IVIM parameter estimation: ballistic regime

Data from the ballistic protocol were used to estimate the IVIM parameters *D*, *f,* and *v*_*d*_ of the ballistic regime by a least-squares fit to data with the following biexponential signal representation:

10 $$S\left( {b,c} \right) = S_{0} \left( {\left( {1 - f} \right)e^{ - bD} + fe^{{ - bD_{b} }} e^{{ - c^{2} v_{d}^{2} }} } \right),$$

where *c* is the flow encoding factor, and *D*_*b*_ and *v*_*d*_ are the diffusion coefficient and velocity dispersion of blood, respectively [8, 12]. To stabilize the fit, *D*_*b*_ was fixed as suggested by Wetscherek at el. [22] with a value of 1.75 µm^2^/ms in accordance with Ahlgren et al. [12]. It is worth noting that Eq. 10 could have been used for processing data from the diffusive protocol as well, but Eq. 9 was used for comparability to the literature.

### Software

All processing of data, unless otherwise stated, was implemented in Python 3.11 with direct calls to packages numpy 1.24, matplotlib 3.7.1, scipy 1.10.1, statsmodels 0.13.5, and nibabel 5.0.1. Project specific functions, including *b*-value optimization, signal drift corrections, and IVIM parameter estimation methods, were gathered in a publicly available python package (https://github.com/oscarjalnefjord/ivim, SHA-1 hash 4f063a1).

### Statistical analysis

For analysis purposes, signal drift was approximated as signal difference between each scan’s first and last *b* = 0 acquisition normalized to the scan duration. Difference in signal drift among brain regions, and difference in IVIM parameter values (both average and difference of repeated scans) between correction methods or *b*-value order were statistically tested using the Friedman test with the exact method for calculating the test statistic (Python package permutation-stats [23]) [24]. If a Friedman test returned a *p*-value < 0.05, pairwise signed-rank tests were employed where p < 0.05 was considered statistically significant.

### Identification of example data

A data set suitable for displaying typical results was identified based on the signal drift in all scans. Data from all subjects, repetitions, regions of interest (ROIs) and scans were ranked based on their distance from the median signal drift of the particular ROI and scan. The subject and repetition with the overall lowest rank was defined as a typical example and used throughout the results section for displaying example data on a subject level.

## Results

### Characterizing the signal drift

**Fig. 3:**
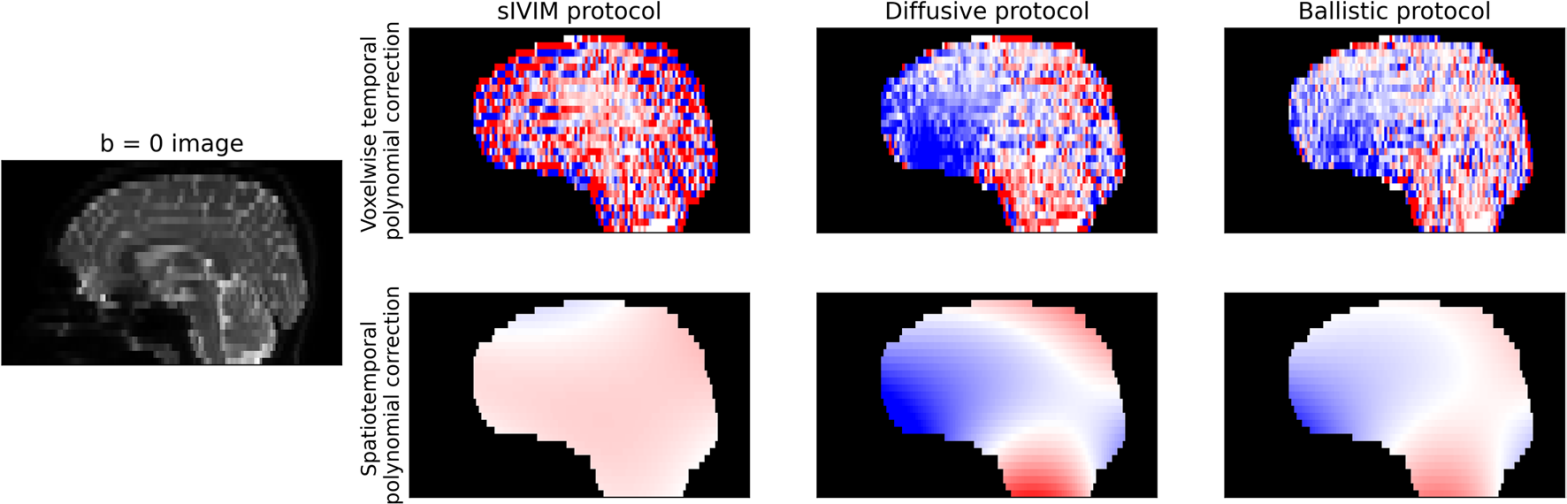
Maps of the spatial distribution of *b* = 0 signal drift (sagittal view) obtained for different protocols (columns) and correction methods (rows) for an example subject. The displayed maps are the correction fields for the last acquisition of each scan with data scaled such that red corresponds to + 10% drift per 5 min, white corresponds to no drift, and blue corresponds to −10% drift per 5 min

A general *b* = 0 signal drift of 1–2% per 5 min was observed when analyzing the whole brain average (blue curves and boxplots in Fig. 2). Significant spatial variations could, however, be seen with typical trends scaling from negative signal drift in the frontal regions of the brain to positive signal drift around the cerebellum (Figs. 2 and 3). Signal drift in the frontal regions was larger than in other parts of the brain with typical drift of around 5% per 5 min (*p* < 0.05).

**Fig. 4:**
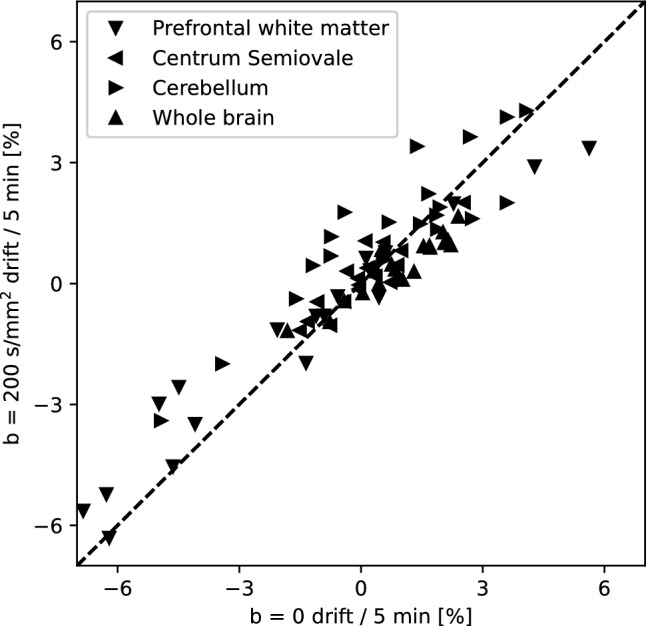
*b* = *0 and b* = *200 s/mm*^*2*^* signal drift in scans with the sIVIM protocol.* Each data point represents the estimated drift for a subject in a given ROI. Displayed data is the slope of a linear fit to the ROI median signal as a function of acquisition time point for each scan. For *b* = 200 s/mm^2^, a random intercept was included in the linear model to account for the directional dependence of the signal. Note that this analysis only was possible for the sIVIM protocol as it required repeated acquisition of the same diffusion encoding direction for non-zero *b*-values (here 200 s/mm^2^). The dashed line shows the unity line

Signal drift at *b* = 200 s/mm^2^ displayed similar trends as those seen at *b* = 0, as shown in Fig. 4 using data acquired with the sIVIM protocol.

### Influence of acquisition order on signal drift effects

**Fig. 5:**
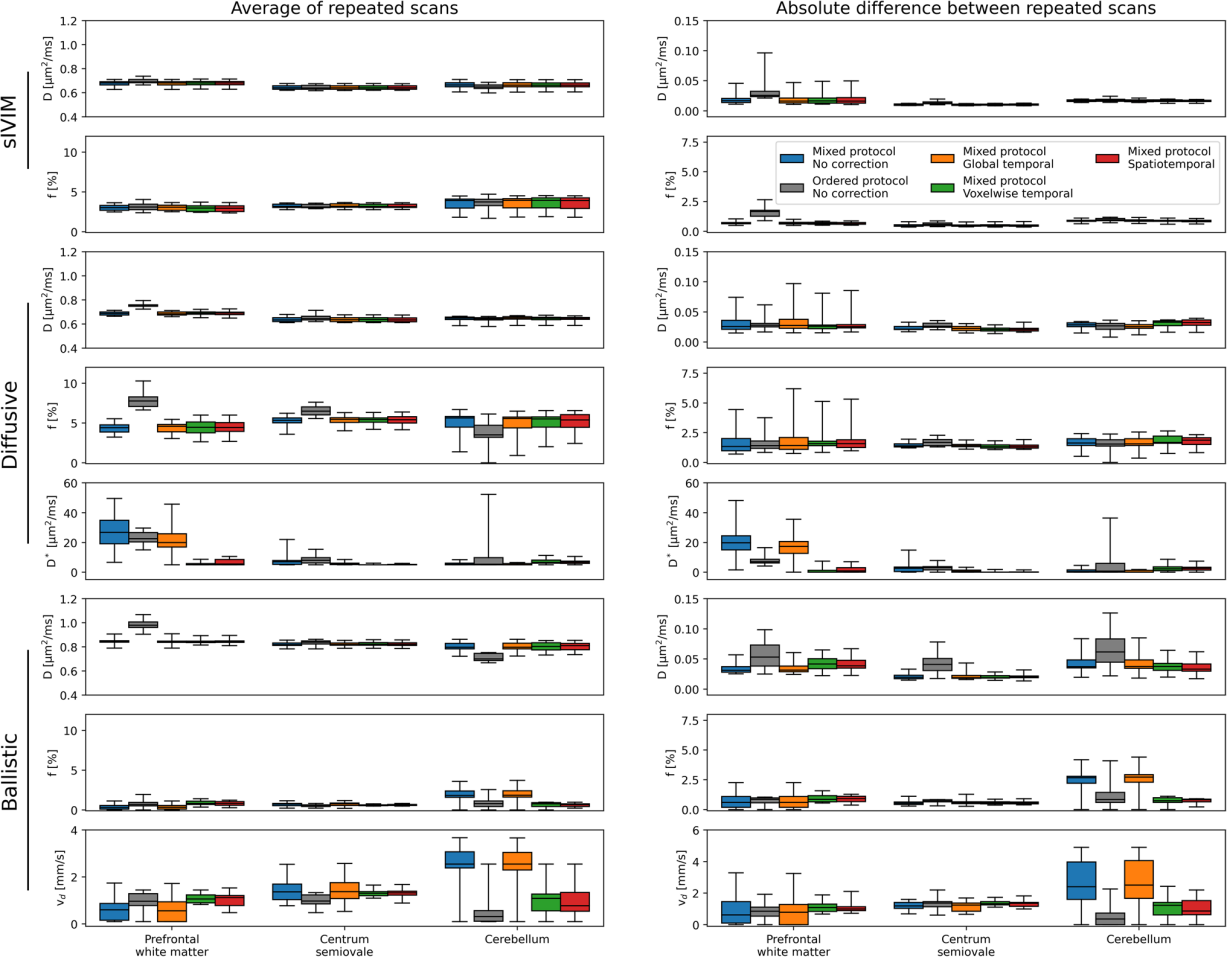
Comparison of estimated IVIM parameter values (left column) and their repeatability (right column) based on data with different acquisition order (mixed/ordered protocol) or using different signal drift correction methods (color legend in right column). Parameters common to all protocols (D and f) share range on *y*-axis to facilitate a more direct comparison among protocols. Note that the left and right columns have different *y*-axis ranges. Boxplots show min, 25th percentile, median, 75th percentile and max. Numerical data is available in Tables 1 and 2, and corresponding example parameter maps are available in Fig. 6

**Fig. 6:**
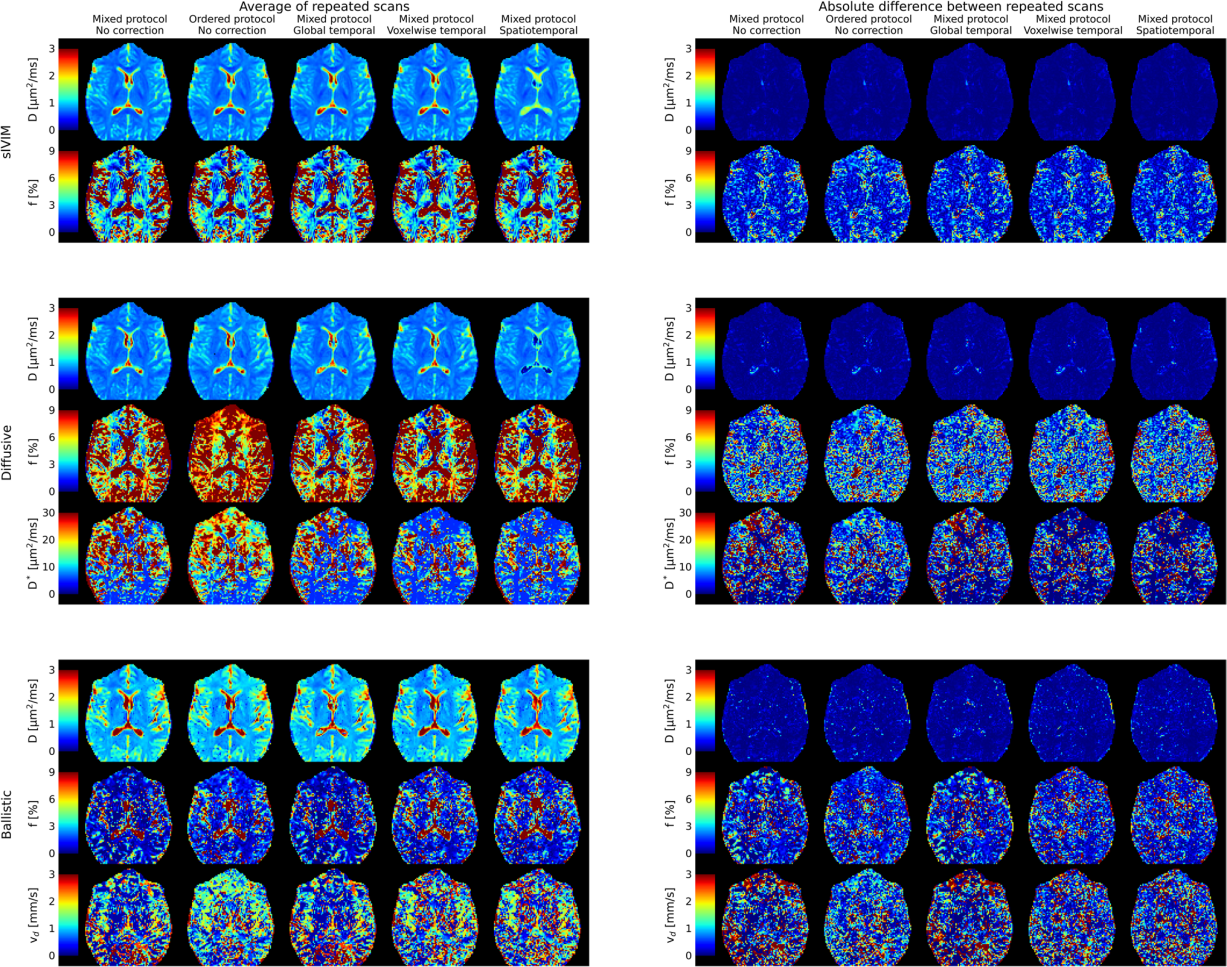
Example IVIM parameter maps (left column) and their repeatability (right column) based on data with different acquisition order (mixed/ordered protocol) or using different signal drift correction methods. Numerical data on the group level is available in Tables 1 and 2, and shown graphically in Fig. 5

Different acquisition orders had different effects on estimated IVIM parameters for different scans/models (Figs. 5 and 6, and Table 1). From the sIVIM protocol, *D* show small but statistically significant differences in prefrontal white matter and cerebellum, while *f* did not show any systematic differences due to acquisition order. Meanwhile, the repeatability of both parameters was lower with the ordered protocol as seen with approximately twice as high difference between repeated scans in the frontal ROI (upper parts of Figs. 5 and 6, and Table 1).

**Table 1:** Effect of acquisition order (mixed or ordered *b*-value order) on IVIM parameter values (average of and difference between repeated scans) using different protocols (sIVIM, diffusive, and ballistic)

	Average of repeated scans		Absolute difference between repeated scans	
	Mixed	Ordered	Mixed	Ordered
sIVIM				
*D* [µm^2^/ms]				
PFWM	**0.68 (0.67–0.69)**^**2**^	**0.69 (0.68–0.71)**^**1**^	**0.02 (0.01–0.02)**^**2**^	**0.03 (0.02–0.03)**^**1**^
CS	0.64 (0.63–0.66)	0.64 (0.63–0.66)	**0.01 (0.01–0.01)**^**2**^	**0.01 (0.01–0.01)**^**1**^
CB	**0.66 (0.65–0.68)**^**2**^	**0.65 (0.64–0.67)**^**1**^	0.02 (0.02–0.02)	0.02 (0.02–0.02)
*f* [%]				
PFWM	3.01 (2.69–3.29)	3.08 (2.77–3.45)	**0.72 (0.62–0.73)**^**2**^	**1.65 (1.27–1.76)**^**1**^
CS	3.16 (3.05–3.46)	3.09 (3.01–3.44)	0.45 (0.42–0.55)	0.58 (0.48–0.70)
CB	3.90 (2.96–4.10)	3.73 (3.26–4.06)	0.90 (0.83–0.92)	0.95 (0.88–1.08)
Diffusive				
*D* [µm^2^/ms]				
PFWM	**0.69 (0.67–0.70)**^**2**^	**0.76 (0.75–0.76)**^**1**^	0.03 (0.02–0.04)	0.03 (0.03–0.03)
CS	**0.63 (0.62–0.65)**^**2**^	**0.65 (0.63–0.66)**^**1**^	**0.02 (0.02–0.03)**^**2**^	**0.03 (0.02–0.03)**^**1**^
CB	**0.65 (0.64–0.66)**^**2**^	**0.64 (0.63–0.65)**^**1**^	**0.03 (0.03–0.03)**^**2**^	**0.03 (0.02–0.03)**^**1**^
*f* [%]				
PFWM	**4.41 (3.87–4.78)**^**2**^	**7.77 (7.08–8.29)**^**1**^	1.34 (0.99–2.01)	1.41 (1.18–1.81)
CS	**5.34 (5.03–5.65)**^**2**^	**6.49 (6.03–7.02)**^**1**^	**1.39 (1.31–1.56)**^**2**^	**1.68 (1.41–1.92)**^**1**^
CB	**5.65 (4.50–5.82)**^**2**^	**3.50 (3.21–4.71)**^**1**^	1.63 (1.43–1.99)	1.54 (1.38–1.90)
D* [µm^2^/ms]				
PFWM	26.84 (19.07–34.92)	22.58 (20.34–26.74)	**19.82 (15.03–24.45)**^**2**^	**7.07 (6.24–8.62)**^**1**^
CS	7.00 (5.24–7.84)	7.98 (6.21–9.82)	2.63 (0.33–3.38)	3.06 (1.54–3.68)
CB	5.22 (5.00–6.03)	5.34 (5.00–9.73)	0.43 (0.00–1.53)	0.68 (0.00–5.93)
Ballistic				
*D* [µm^2^/ms]				
PFWM	**0.85 (0.84–0.85)**^**2**^	**0.98 (0.96–1.01)**^**1**^	0.03 (0.03–0.04)	0.05 (0.04–0.07)
CS	0.82 (0.81–0.83)	0.84 (0.82–0.85)	**0.02 (0.02–0.02)**^**2**^	**0.04 (0.03–0.05)**^**1**^
CB	**0.80 (0.78–0.83)**^**2**^	**0.70 (0.69–0.75)**^**1**^	0.04 (0.04–0.05)	0.06 (0.04–0.08)
*f* [%]				
PFWM	0.31 (0.10–0.55)	0.68 (0.50–0.94)	0.59 (0.19–1.09)	0.85 (0.55–0.97)
CS	0.73 (0.53–0.81)	0.49 (0.43–0.63)	0.53 (0.42–0.63)	0.71 (0.64–0.82)
CB	1.81 (1.57–2.38)	0.79 (0.43–1.11)	2.66 (2.20–2.79)	0.84 (0.59–1.44)
*v*_*d*_ [mm/s]				
PFWM	0.60 (0.15–0.87)	0.97 (0.78–1.29)	0.62 (0.10–1.46)	0.85 (0.54–1.11)
CS	1.37 (1.04–1.69)	0.98 (0.85–1.24)	1.20 (1.03–1.41)	1.38 (1.14–1.46)
CB	**2.55 (2.38–3.07)**^**2**^	**0.31 (0.12–0.57)**^**1**^	**2.41 (1.60–3.97)**^**2**^	**0.36 (0.00–0.74)**^**1**^

The superscripts 1–2 indicate statistically significant differences (*p* < 0.05) relative to analysis of data with acquisition order *i* (mixed *i* = 1, ordered *i* = 2). Values are reported as group median (25th percentile—75th percentile)

*PFWM* prefrontal white matter, *CS* centrum semiovale, *CB* cerebellum

*D* and *f* obtained with the diffusive protocol did instead display the opposite behavior with systematic differences between the ordered and mixed protocols in the frontal ROI (about 10% for *D* and nearly 100% for *f*), while the repeatability was affected to a smaller extent by choice of protocol (middle parts of Figs. 5 and 6, and Table 1). On the other hand, D* obtained from the same scan showed no distinct systematic differences due to acquisition order, but a higher repeatability for the ordered protocol manifested as a halved difference between repeated scans in the frontal ROI.

The acquisition order tended to introduce both systematic differences and to affect repeatability for the IVIM parameters obtained with the ballistic protocol (lower parts of Figs. 5 and 6, and Table 1). *D* was about 15% higher in the frontal ROI and equally lower in the cerebellum ROI for the ordered protocol relative to the mixed protocol. The repeatability of *D* was also approximately a factor two better in all ROIs with the mixed protocol. *f* and *v*_*d*_ showed similar trends with systematically higher estimates and differences between repeated scans in the cerebellum ROI using the mixed protocol, although *f* did not reach statistical significance.

**Fig. 7:**
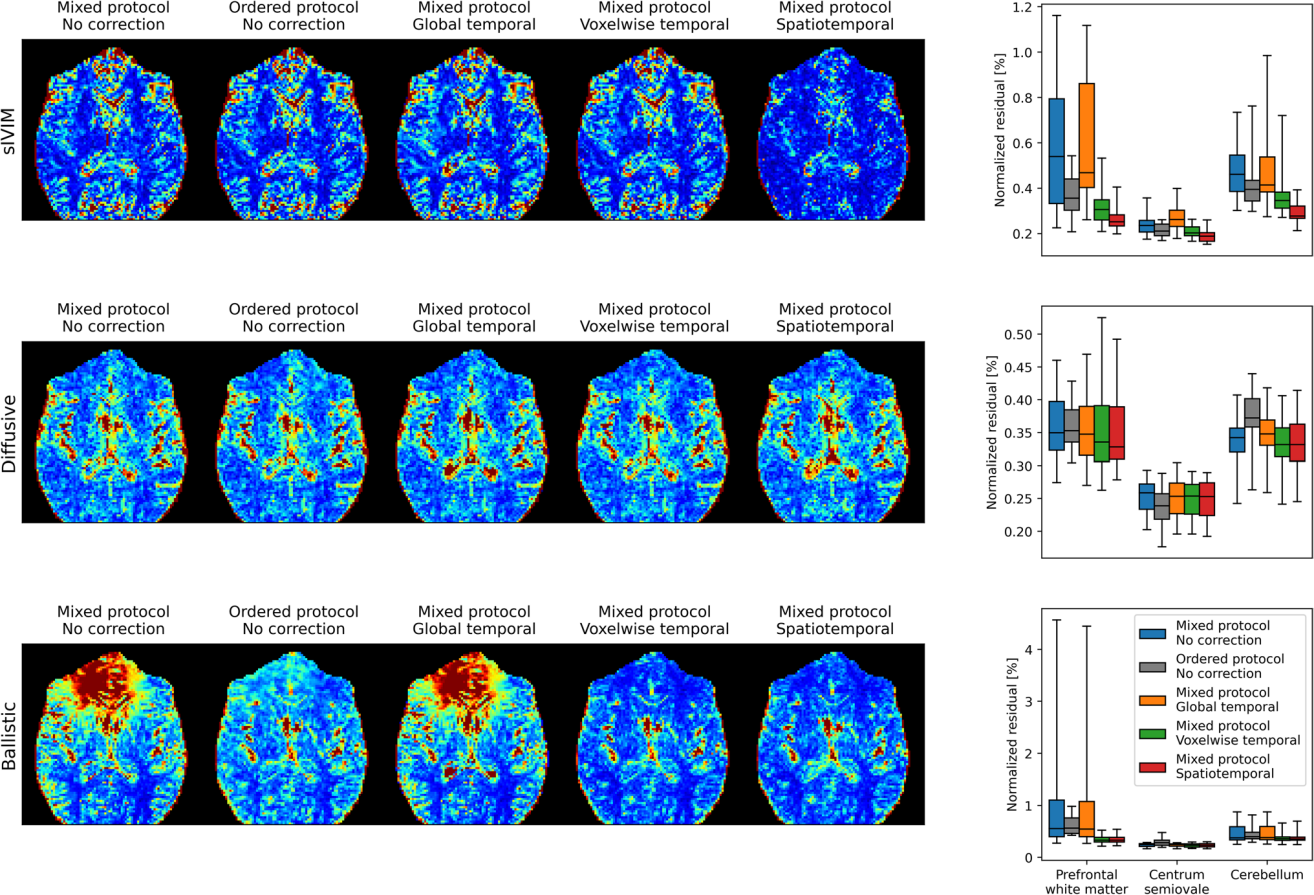
*Comparison of residuals among different acquisition orders and correction methods.* Residuals were calculated as the root mean squared difference between the model fits and the acquired data. Example residual maps are shown to the left with an arbitrary scale that still allows for comparison among different acquisition orders and correction methods. Group summaries are shown to the left for the previously investigated ROIs. Boxplots show min, 25th percentile, median, 75th percentile, and max

The fit to data was only mildly affected by acquisition order, except for some scans with the ballistic protocols with very large residuals in the frontal ROI for the mixed protocol relative to the ordered protocol (Fig. 7).

### Correcting for signal drift

The global temporal polynomial correction was, in general, only able to reduce signal drift observed on a whole-brain level (yellow curves and boxplots in Fig. 2). The voxelwise temporal and spatiotemporal polynomial correction did, on the other hand, consistently minimize the signal drift (green and red curves and boxplots, respectively, in Fig. 2). The correction fields generated by the voxelwise temporal and spatiotemporal polynomial corrections showed similar spatial patterns (Fig. 3).

Choice of correction method only resulted in distinct systematic changes in parameter estimates or repeatability in a few cases (Figs. 5 and 6, and Table 2). Estimates of D* and its absolute difference between repeated scans were substantially lower with the voxelwise temporal or spatiotemporal polynomial corrections, in particular in the frontal region. *f* and *v*_*d*_ obtained with the ballistic protocol tended to give more similar parameter estimates and repeatability across ROIs when voxelwise temporal or spatiotemporal polynomial correction was applied, as opposed to the lower/higher estimates and the difference between repeated scans seen in the frontal/cerebellum ROIs with no or global temporal polynomial correction.

**Table 2:** Effect of signal drift correction method (no, global temporal, voxelwise temporal or spatiotemporal correction) on IVIM parameter values (average of and difference between repeated scans) using different protocols (sIVIM, diffusive, and ballistic)

	Average of repeated scans				Absolute difference between repeated scans			
	No correction	Global	Voxelwise	Spatiotemporal	No correction	Global	Voxelwise	Spatiotemporal
sIVIM								
*D* [µm^2^/ms]								
PFWM	**0.68 (0.67–0.69)**^**2,3,4**^	**0.68 (0.66–0.69)**^**1,3,4**^	**0.68 (0.67–0.69)**^**1,2**^	**0.68 (0.67–0.69)**^**1,2**^	0.02 (0.01–0.02)	0.02 (0.01–0.02)	0.02 (0.01–0.02)	0.02 (0.01–0.02)
CS	0.64 (0.63–0.66)	0.64 (0.63–0.66)	0.64 (0.63–0.66)	0.64 (0.63–0.66)	0.01 (0.01–0.01)	0.01 (0.01–0.01)	0.01 (0.01–0.01)	0.01 (0.01–0.01)
CB	**0.66 (0.65–0.68)**^**2,3**^	**0.66 (0.65–0.68)**^**1**^	**0.66 (0.65–0.68)**^**1**^	**0.66 (0.65–0.68)**	0.02 (0.02–0.02)	0.02 (0.02–0.02)	0.02 (0.02–0.02)	0.02 (0.02–0.02)
*f* [%]								
PFWM	**3.01 (2.69–3.29)**^**2,3,4**^	**3.07 (2.69–3.33)**^**1,3,4**^	**2.97 (2.53–3.26)**^**1,2**^	**2.94 (2.53–3.24)**^**1,2**^	0.72 (0.62–0.73)	0.67 (0.62–0.75)	0.68 (0.59–0.74)	0.70 (0.60–0.74)
CS	**3.16 (3.05–3.46)**^**2**^	**3.20 (3.08–3.50)**^**1,3**^	**3.16 (3.05–3.46)**^**2**^	**3.15 (3.05–3.44)**	0.45 (0.42–0.55)	0.45 (0.43–0.53)	0.45 (0.43–0.54)	0.46 (0.42–0.54)
CB	**3.90 (2.96–4.10)**^**2,3**^	**3.93 (2.99–4.15)**^**1**^	**3.94 (2.99–4.25)**^**1,4**^	**3.93 (2.92–4.26)**^**3**^	0.90 (0.83–0.92)	0.88 (0.83–0.96)	0.89 (0.83–0.93)	0.89 (0.81–0.93)
Diffusive								
*D* [µm^2^/ms]								
PFWM	0.69 (0.67–0.70)	0.68 (0.67–0.70)	0.69 (0.68–0.70)	0.69 (0.68–0.70)	0.03 (0.02–0.04)	0.03 (0.02–0.04)	0.03 (0.02–0.03)	0.03 (0.02–0.03)
CS	0.63 (0.62–0.65)	0.63 (0.62–0.65)	0.64 (0.62–0.65)	0.63 (0.62–0.65)	**0.02 (0.02–0.03)**^**3**^	**0.02 (0.02–0.03)**	**0.02 (0.02–0.02)**^**1**^	**0.02 (0.02–0.02)**
CB	0.65 (0.64–0.66)	0.65 (0.64–0.66)	0.65 (0.63–0.65)	0.65 (0.64–0.65)	**0.03 (0.03–0.03)**^**2,3,4**^	**0.03 (0.02–0.03)**^**1,3,4**^	**0.03 (0.03–0.04)**^**1,2**^	**0.03 (0.03–0.04)**^**1,2**^
*f* [%]								
PFWM	4.41 (3.87–4.78)	4.58 (3.89–4.83)	4.47 (3.77–5.12)	4.44 (3.94–5.04)	1.34 (0.99–2.01)	1.43 (1.10–2.09)	1.59 (1.41–1.79)	1.58 (1.26–1.90)
CS	5.34 (5.03–5.65)	5.48 (5.08–5.66)	5.43 (5.11–5.66)	5.40 (4.99–5.80)	1.39 (1.31–1.56)	1.41 (1.32–1.52)	1.32 (1.20–1.48)	1.33 (1.20–1.45)
CB	5.65 (4.50–5.82)	5.57 (4.37–5.75)	5.50 (4.49–5.78)	5.38 (4.47–6.04)	1.63 (1.43–1.99)	1.57 (1.43–2.00)	1.69 (1.62–2.22)	1.88 (1.51–2.08)
D* [µm^2^/ms]								
PFWM	**26.84 (19.07–34.92)**^**2,3,4**^	**19.95 (16.88–25.91)**^**1,3,4**^	**5.11 (5.00–5.74)**^**1,2**^	**5.45 (5.00–8.53)**^**1,2**^	**19.82 (15.03–24.45)**^**3,4**^	**17.36 (12.68–20.71)**^**3,4**^	**0.02 (0.00–1.17)**^**1,2**^	**0.84 (0.00–2.99)**^**1,2**^
CS	**7.00 (5.24–7.84)**^**2,3,4**^	**5.35 (5.02–6.03)**^**1,3,4**^	**5.00 (5.00–5.00)**^**1,2**^	**5.04 (5.00–5.15)**^**1,2**^	**2.63 (0.33–3.38)**^**2,3,4**^	**0.56 (0.04–1.35)**^**1,3,4**^	**0.00 (0.00–0.00)**^**1,2**^	**0.04 (0.00–0.15)**^**1,2**^
CB	**5.22 (5.00–6.03)**^**3,4**^	**5.00 (5.00–5.78)**^**3,4**^	**6.65 (5.98–8.09)**^**1,2**^	**6.77 (5.92–7.44)**^**1,2**^	**0.43 (0.00–1.53)**^**3,4**^	**0.00 (0.00–1.26)**^**3,4**^	**2.23 (0.95–3.57)**^**1,2**^	**2.40 (1.29–3.27)**^**1,2**^
Ballistic								
*D* [µm^2^/ms]								
PFWM	0.85 (0.84–0.85)	0.85 (0.84–0.85)	0.84 (0.84–0.85)	0.84 (0.84–0.85)	**0.03 (0.03–0.04)**^**3,4**^	**0.03 (0.03–0.04)**^**3**^	**0.04 (0.03–0.05)**^**1,2**^	**0.04 (0.03–0.05)**^**1**^
CS	0.82 (0.81–0.83)	0.82 (0.81–0.83)	0.82 (0.81–0.84)	0.82 (0.81–0.83)	0.02 (0.02–0.02)	0.02 (0.02–0.02)	0.02 (0.02–0.02)	0.02 (0.02–0.02)
CB	0.80 (0.78–0.83)	0.80 (0.78–0.83)	0.80 (0.78–0.84)	0.81 (0.78–0.83)	0.04 (0.04–0.05)	0.04 (0.03–0.05)	0.04 (0.03–0.04)	0.03 (0.03–0.04)
*f* [%]								
PFWM	**0.31 (0.10–0.55)**^**3,4**^	**0.32 (0.10–0.55)**^**3,4**^	**0.82 (0.67–1.09)**^**1,2**^	**0.81 (0.59–1.03)**^**1,2**^	0.59 (0.19–1.09)	0.59 (0.19–1.10)	0.85 (0.65–1.16)	0.93 (0.66–1.11)
CS	0.73 (0.53–0.81)	0.75 (0.56–0.87)	0.56 (0.52–0.63)	0.58 (0.55–0.67)	0.53 (0.42–0.63)	0.55 (0.49–0.64)	0.56 (0.48–0.65)	0.57 (0.47–0.64)
CB	**1.81 (1.57–2.38)**^**2,3,4**^	**1.83 (1.59–2.41)**^**1,3,4**^	**0.75 (0.42–0.91)**^**1,2**^	**0.66 (0.43–0.77)**^**1,2**^	**2.66 (2.20–2.79)**^**2,3,4**^	**2.73 (2.30–2.94)**^**1,3,4**^	**0.73 (0.59–0.99)**^**1,2**^	**0.81 (0.65–0.87)**^**1,2**^
*v*_*d*_ [mm/s]								
PFWM	**0.60 (0.15–0.87)**^**3**^	**0.56 (0.10–0.94)**^**3**^	**1.06 (0.90–1.23)**^**1,2**^	**1.13 (0.78–1.21)**	0.62 (0.10–1.46)	0.79 (0.00–1.28)	1.09 (0.84–1.31)	1.00 (0.89–1.14)
CS	1.37 (1.04–1.69)	1.37 (1.08–1.75)	1.31 (1.20–1.37)	1.32 (1.23–1.39)	1.20 (1.03–1.41)	1.24 (0.86–1.37)	1.33 (1.26–1.46)	1.35 (1.16–1.41)
CB	**2.55 (2.38–3.07)**^**3,4**^	**2.55 (2.29–3.04)**^**3,4**^	**1.09 (0.55–1.27)**^**1,2**^	**0.77 (0.54–1.34)**^**1,2**^	2.41 (1.60–3.97)^3,4^	2.51 (1.67–4.06)^3,4^	1.23 (0.62–1.41)^1,2^	0.87 (0.63–1.53)^1,2^

The superscripts 1–4 indicate statistically significant differences (*p* < 0.05) relative to analysis of data with correction method *i* (no *i* = 1, global *i* = 2, voxelwise *i* = 3, spatiotemporal *i* = 4). Values are reported as group median (25th percentile—75th percentile)

*PFWM* Prefrontal white matter, *CS* Centrum semiovale, *CB* Cerebellum

The fit to data tended to be as good or better when using voxelwise temporal or spatiotemporal polynomial corrections (Fig. 7). The residuals of the fit to Eq. 10 (ballistic model) showed similar trends as those for the sIVIM fit but with some scans having particularly large residuals in the frontal ROI with no or only a global temporal polynomial correction.

## Discussion

The results of this study emphasize the importance of taking systematic signal drift into account when analyzing dMRI data of the human brain. The observed signal drift was around 2% per 5 min in the brain in general but reached above 5% per 5 min in the frontal regions, indicating the need for spatially varying correction methods. Use of such methods resulted in improved model fitting and to some extent better repeatability. The different effects of signal drift on dMRI parameter estimation, depending on the order in which data is acquired, were also demonstrated. Furthermore, error propagation had the effect that signal drifts of a few or some percent, for some dMRI parameters, could introduce errors on the same order of magnitude as the actual model parameter values.

Whole-brain signal drift of approximately 2% per 5 min as observed in the current study agrees well with the 5% over a 15 min scan reported by Vos et al. [2]. Spatially varying signal drift was reported in phantoms by Hansen et al. [6] and in a single subject by Huynh et al. [7] similar to the results of the current study. However, the actual patterns and levels are not directly comparable as Hansen et al. acquired all data in phantoms while Huynh et al. did not report total scan time. The current study adds to the evidence that correction for signal drift in dMRI is of importance, showing particularly large effects in the frontal regions of the brain.

Given the spatially dependent signal drift, a correction method beyond a global correction working on signal averaged across the whole brain is needed. The results of this study indicate that the ability to correct for spatially varying signal drift appears to be similar for voxelwise temporal and spatiotemporal polynomial correction, suggesting that a second order polynomial is sufficient to approximate spatial signal drift variations in the human brain. Depending on noise level in the particular data set of interest, the spatiotemporal polynomial correction may be preferable as it can be assumed to be less sensitive to noise given the parameterized form of the estimated correction field. The underlying assumption—for all evaluated correction methods—that the signal drift observed at *b* = 0 is an indicator of the signal drift for all *b*-values could be confirmed in Fig. 4, verifying the suitability of such methods.

The importance of correcting for signal drift in dMRI of the brain increases with scan time and is thus of more central concern in a research setting where longer scans are more common. While most dMRI scans conducted in clinical practice are short, typically only aiming for quantification of the apparent diffusion coefficient, some scans are longer, for example those used to generate tractographies for neurosurgical planning. In these cases, corrections for signal drift could be of importance to improve data quality. Furthermore, as use of dMRI methods like diffusion kurtosis imaging or IVIM increases in the clinic [8, 9], longer scan times where signal drift becomes noticeable will be more common, thus increasing the need for suitable correction methods.

The underlying source of the observed signal drift remains unknown, but the high similarity between the estimated correction fields and typical off-resonance fields suggests that parts of the signal drift may be due to varying off-resonance effects [25]. This may be alleviated by dynamic shimming, but unless a very time efficient procedure can be implemented, a retrospective correction, as used in the current study, might be preferable to not increase the overall scan time.

The effect of signal drift on IVIM parameter estimation depended on the protocol and model used. The biexponential model used to describe IVIM in the diffusive regime (Eq. 9) is very flexible in nature and can often adapt to the altered signal behavior in the presence of signal drift, as manifested in relatively small fitting residuals and mainly systematic parameter errors. The comparatively more rigid sIVIM (3 *b*-value fit to Eq. 5–8) and IVIM in the ballistic regime (bipolar encoding with/without flow compensation with fit to Eq. 10) did instead produce larger fitting residuals due to signal drift and had both systematic effects and altered repeatability. These expected trends can, to some extent, be used to predict the impact of signal drift on other dMRI models.

Retrospective corrections for signal drift in dMRI, as those evaluated in this study, rely on acquisition schemes with interspersed *b* = 0 images. Furthermore, distributing not only the *b* = 0 acquisitions but all *b*-values and encoding directions throughout the scan can lessen the demands on the gradient hardware and will reduce the systematic effects of signal drift even before any correction is applied. Use of such protocols does, however, typically require imports of custom text files to the MR scanner meaning that its use in clinical practice is limited. A remedy to this issue would be vendors providing an option to distribute *b*-values and encoding directions in some predefined way to open up this option also for non-specialized users. This can be achieved by simply introducing a binary parameter to the user interface that indicates if the default looping structure or one similar to the one used in the current study should be used. Such a looping algorithm can be designed independently of the particular *b*-values and diffusion encoding directions and could thus generalize to any protocol.

The study has some limitations, including a relatively small number of subjects, a single vendor, and a limited number of evaluated dMRI protocols and models. The trends in data are, however, distinct and the presence of signal drift in dMRI has been manifested for multiple vendors previously [2]. The need for corrections for signal drift when using other dMRI protocols and models than those in the current study can be evaluated for each particular implementation, but these results may give an indication in many cases as the protocols and models span from very simple protocols and rigid models to more advanced multidimensional acquisitions [26].

In conclusion, signal drift in dMRI of the brain was observed to be around 2% per 5 min in the brain as a whole, but reached above 5% per 5 min in the frontal regions. This signal drift could be corrected for by using spatially varying polynomial correction methods if data with interspersed *b* = 0 images were acquired. Such corrections appear necessary for scans longer than a few minutes. Errors introduced by signal drift resulted in either biased IVIM parameter estimates, altered repeatability, or both, depending on the particular protocol and model used. D* from the diffusive IVIM model, and the perfusion-related parameters *f* and *v*_*d*_ from the ballistic model were those most strongly affected by signal drift corrections. All IVIM parameters from all evaluated models were to some extent affected by the acquisition order, emphasizing the importance of designing acquisition protocols robust against signal drift.

## Data Availability

Examcards and gradient waveforms, as well as scripts used to process data and to generate figures are available at https://github.com/oscarjalnefjord/publications.

## Appendix A

The second order spatial polynomials used for the spatiotemporal signal drift correction (Eqs. 3 and 4) had the following form:

11 $$p_{i} \left( {x,y,z} \right) = k_{i,0} + k_{i,1} x + k_{i,2} y + k_{i,3} z + k_{i,4} xy + k_{i,5} xz + k_{i,6} yz + k_{i,7} xyz + k_{i,8} x^{2} + k_{i,9} y^{2} + k_{i,10} z^{2} + k_{i,11} x^{2} y + k_{i,12} x^{2} y^{2} + k_{i,13} x^{2} z + k_{i,14} x^{2} z^{2} + k_{i,15} x^{2} yz + k_{i,16} x^{2} y^{2} z + k_{i,17} x^{2} yz^{2} + k_{i,18} x^{2} y^{2} z^{2} + k_{i,19} xy^{2} + k_{i,20} y^{2} z + k_{i,21} y^{2} z^{2} + k_{i,22} xy^{2} z + k_{i,23} xy^{2} z^{2} + k_{i,24} xz^{2} + k_{i,25} yz^{2} + k_{i,26} xyz^{2} ,$$

where $k_{i,j}$ are the polynomial coefficients to estimate.
